# Mitigating work conditions that can inhibit learning from errors: Benefits of error management climate perceptions

**DOI:** 10.3389/fpsyg.2023.1033470

**Published:** 2023-01-20

**Authors:** Oscar van Mourik, Therese Grohnert, Anna Gold

**Affiliations:** ^1^Department of Accounting, School of Business and Economics, Vrije Universiteit Amsterdam, Amsterdam, Netherlands; ^2^Department of Educational Research and Development, School of Business and Economics, Maastricht University, Maastricht, Netherlands

**Keywords:** learning from errors, error management climate, error consequences, error type, emotions, time pressure

## Abstract

**Introduction:**

Professionals do not always learn from their errors; rather, the way in which professionals experience errors and their work environment may not foster, but can rather inhibit error learning. In the wake of a series of accounting scandals, including Royal Ahold in Netherlands, Lehman Brothers in the United States, and Wirecard in Germany, within the context of financial auditing, we explore four audit-specific conditions at the workplace that could be negatively associated with learning: small error consequences, routine-type errors, negative emotions, and high time pressure. Then, we examine how perceptions of an open or blame error management climate (EMC) moderate the negative relationship between the four work conditions and learning from errors.

**Methods:**

Using an experiential questionnaire approach, we analyze data provided by 141 Dutch auditors across all hierarchical ranks from two audit firms.

**Results:**

Our results show that open EMC perceptions mitigate the negative relationship between negative emotions and error learning, as well as the negative relationship between time pressure and error learning. While we expected that blame EMC perceptions would exacerbate the negative relationship between negative emotions and error learning, we find a mitigating effect of low blame EMC perceptions. Further, and contrary to our expectations, we find that blame EMC perceptions mitigate the negative relationship between small error consequences and error learning, so that overall, more error learning takes place regardless of consequences when participants experience a blame EMC. *Post-hoc* analyses reveal that there is in fact an inverted- U-shaped relationship between time pressure and error learning.

**Discussion:**

We derive several recommendations for future research, and our findings generate specific implications on how (audit) organizations can foster learning from errors.

## 1. Introduction

Human errors at work are unavoidable, even when organizations develop sophisticated systems for their prevention (van Dyck et al., 2005; Frese and Keith, 2015; Metcalfe, 2017). Efforts to prevent planes from crashing and patients from dying have had a significant impact, yet are unable to fully guard against errors at work (Ely et al., 2011; Hagen, 2013; Seckler et al., 2017). Consequently, effective error management in organizations requires both prevention and subsequent learning from errors that do occur (van Dyck et al., 2005), defined as “the process through which individuals (a) reflect on errors that they have made, (b) locate the root causes of the errors, (c) develop knowledge about action–outcome relationships and the effects of these relationships on the work environment, and (d) use this knowledge to modify or improve their behavior or decision making” (Zhao, 2011, p. 436). In this study, we explore error learning in the context of financial statement auditing. Auditors assess whether organizations have reported their financial statements fairly and in line with international reporting standards, a complex task that is carried out in hierarchical teams. Auditors’ judgments of a client’s financial statements are communicated to the wider public, such as governments, investors, and other stakeholders. Over the last decades, the domain of auditing has experienced a series of scandals, such as Royal Ahold in Netherlands, Lehman Brothers in the United States, and Wirecard in Germany, resulting in tighter regulation, public oversight, and public shaming of key actors (i.e., Storbeck, 2020; Rotteveel, 2022). Consequently, audit firms are investing significantly in procedures that foster both error prevention and subsequent learning from errors (e.g., ICAEW, 2016; KPMG, 2016; FRC, 2021). Meanwhile, research on error learning in auditing is still scarce (i.e., Gold et al., 2022; Smeets et al., 2022).

Learning from errors does not occur spontaneously; rather, it is an effortful process that requires time, resources, and vulnerability, which may not always be available or desirable in the workplace (Zhao, 2011; Lei et al., 2016; Metcalfe, 2017). Prior error learning research indicates that individual learning from errors depends on whether conditions at work are perceived by individuals to positively or negatively affect their self-worth and well-being (Kanfer and Ackerman, 1989; Edmondson, 2004; Tulis et al., 2016). Building on insights from an interview study with auditors on error management by Gold et al. (2022), we explore four work conditions that could inhibit auditors’ error learning in daily practice: small consequences for errors, routine-type errors, strong negative emotions, and high time pressure. First, we expect that auditors will be less likely to engage in error learning when an error has smaller (rather than larger) error consequences (in line with, i.e., Levitt and March, 1988; Cannon and Edmondson, 2005; Homsma et al., 2009). That is, errors with smaller consequences can be considered less ‘learn-worthy’; however, ignoring their learning potential may lead to repetition and escalation in the future (Cannon and Edmondson, 2005). Second, we hypothesize that auditors will report less error learning from routine (compared to non-routine) errors (in line with Embrey, 2005; Zhang et al., 2019), as these errors are easily attributable to inattention or coincidence, rather than a lack of knowledge (Sutcliffe and Rugg, 1998). Third, we hypothesize that auditors will learn less from errors when they experience strong (rather than weak) negative emotions, as these emotions take up crucial cognitive resources needed for learning, and may inhibit error learning (in line with, i.e., Heimbeck et al., 2003; Zhao et al., 2019; Smeets et al., 2022). Finally, we expect that auditors will report less error learning with high (rather than low) time pressure, as they are likely to prioritize urgent tasks over learning, and because the error’s cause can be externalized (in line with, i.e., Zhao and Olivera, 2006; Homsma et al., 2009; Putz et al., 2012).

To date, most research focuses on conditions that *foster* error learning (Rybowiak et al., 1999; van Dyck et al., 2005; Bligh et al., 2018). By exploring work conditions that may *inhibit* error learning, we contribute to extant research by identifying where organizations can intervene to create necessary conditions for enabling error learning. To this end, we examine how auditors’ error management climate (EMC) perceptions interact with the four work conditions with regard to error learning. EMC describes the beliefs, norms, and practices related to how errors are dealt with that are shared within an organization (van Dyck et al., 2005). An open EMC promotes opportunities for learning from errors. We expect that the negative effect of the aforementioned conditions on error learning will be mitigated (i.e., weakened) when auditors perceive that they work in an open EMC. On the other hand, the negative effect of the work conditions on learning will be exacerbated (i.e., strengthened) when auditors work in a perceived blame EMC, where errors are typically seen as personal failures and are therefore punished (van Dyck et al., 2005). By studying the interaction between EMC perceptions and the four conditions with regard to error learning, we contribute to extant research on the direct relationship between EMC and error learning (for reviews, see Lei et al., 2016; Metcalfe, 2017). We also provide novel insights into how organizations can mitigate the negative impact of the four work conditions on error learning.

## 2. Hypothesis development

In their interview study on error management in auditing, Gold et al. (2022) reveal that in the wake of significant pressure on the profession, audit firms predominantly focus on error prevention, rather than fully embracing the learning potential of errors to improve their performance. Practitioners describe a series of work conditions that appear to influence error learning, such as the potential consequences of errors for the client, the types of errors dealt with, and experiencing emotions in connection with errors, along with experienced time pressure. In this study, we explore how these four work conditions relate to error learning by building hypotheses that follow prior research.

### 2.1. Work conditions inhibiting error learning

#### 2.1.1. Small error consequences

First, auditors, like other professionals, make errors that vary in their *consequences* (Gold et al., 2022). While all errors, regardless of their consequences, carry learning potential (van Dyck, 2009), prior research has shown that learning is more likely to occur when an error has relatively larger consequences, usually affecting the person committing the error or others, with regard to individuals’ health, finances, or social standing (Cannon and Edmondson, 2005; Homsma et al., 2009). These errors challenge the existing state of affairs and stimulate individuals to engage in learning to prevent these significant consequences (Levitt and March, 1988). Meanwhile, professionals typically fail to learn from errors with relatively minor consequences (Baumard and Starbuck, 2005). Despite their theoretical learning potential, errors with smaller consequences are more easily discounted as irrelevant because, as shown by Baumard and Starbuck (2005), professionals focus on achieving expected outcomes; errors that do not significantly affect these outcomes are not made a priority for learning (see also Levitt and March, 1988; Cannon and Edmondson, 2005). Hence, failing to learn from errors with minor consequences may have serious long-term implications. Following these insights from prior research, we formulate our first hypothesis:

*Hypothesis* 1a: Professionals will report less error learning when they experience small (rather than large) error consequences.

#### 2.1.2. Routine errors

Second, professionals may not deem all *error types* to be equally ‘learn-worthy’. Within organizational psychology, the influential classification of error types by Rasmussen (1986) distinguishes between errors that are routine and non-routine. On the routine side, skills-based errors occur during recurring and predictable situations and typically result from lack of attention/memory, while knowledge to solve the problem is technically present and available (in line with Sutcliffe and Rugg, 1998; Zhang et al., 2019). For example, a professional may forget to attach a document to an email when in a hurry. Non-routine errors occur either when the necessary knowledge to solve a problem is present but not used correctly, or when a task is so complex and/or unfamiliar that new knowledge is needed to address the situation (respectively known as rules-based and knowledge-based errors; Sutcliffe and Rugg, 1998; Zhang et al., 2019), for example, applying a checklist or decision rule in the wrong context. The wider error learning literature shows that with non-routine errors, individuals are typically challenged to create understanding of complex contextual information related to the error, forcing them to reconsider the applicability and limitations of their knowledge, activities naturally linked to learning (Rasmussen, 1986; Embrey, 2005). In contrast, routine errors are often ignored, as they are not perceived to be related to knowledge, and hence, the need to learn (Embrey, 2005). In this study, we therefore hypothesize the following:

*Hypothesis* 1b: *Professionals will report less error learning when they experience routine (rather than non-routine) errors.*

#### 2.1.3. Strong negative emotions

Third, *emotions* that individuals experience when discovering they made an error may also inhibit error learning (Zhao, 2011; Rausch et al., 2017). Past research has established that experiencing strong negative emotions such as shame, fear, or guilt can limit learning for two principal reasons. First, experiencing these emotions may cause professionals to withdraw from the situation, missing out on important learning opportunities (Zhao et al., 2019). Second, these emotions occupy a person’s working memory, limiting the information processing that is essential for leaning (Zhao, 2011; Tulis et al., 2016; Smeets et al., 2022). Literature on emotional regulation has shown that individuals prioritize relieving strong emotions over problem-solving and learning, with the consequence that strong negative emotions may inhibit error learning (Kanfer and Ackerman, 1989; Ilgen and Davis, 2000; Tulis et al., 2016; Hökkä et al., 2020). Yet, studies show that when individuals succeed in tempering these negative emotions, they free up mental space to adopt a more accepting view of errors, resulting in a stronger motivation to learn (van Dyck et al., 2005; Frese and Keith, 2015). Indeed, experimental research shows that individuals are more effective in learning from errors when they do not experience strong negative emotions (Heimbeck et al., 2003), leading us to the following:

*Hypothesis* 1c: Professionals will report less error learning when they experience stronger (rather than weaker) negative emotions related to the error.

#### 2.1.4. High time pressure

Fourth, auditors frequently experience *time pressure* (Pierce and Sweeney, 2004; Gold et al., 2022), rooted in the cyclical and commercial nature of auditing. Auditors are faced with strict filing deadlines, as an audit becomes more profitable when auditors use fewer hours than paid for upfront by the client (i.e., Kelley and Margheim, 1990; DeZoort and Lord, 1997). Several organizational behavior studies suggest that contextual factors causing time pressure can impair learning from errors for two main reasons (Zhao and Olivera, 2006; Homsma et al., 2009; Putz et al., 2012). First, when individuals attribute errors to external causes such as time pressure, it is less likely that they will recognize the opportunity to learn, as they first seek closure (Ellis and Davidi, 2005; Putz et al., 2012). While such a strategy enables them to meet important deadlines, it may be problematic for learning because it leads to superficial error analysis, at best (Kruglanski et al., 1993; Putz et al., 2012). Second, high time constraints may cause individuals to use information-processing strategies that limit their cognitive capability (Zhao and Olivera, 2006), which is an essential element of learning from errors (Rasmussen, 1986; Metcalfe, 2017). Both Ford et al. (1989) and Zhao and Olivera (2006) found that professionals ignore competing hypotheses and filter out contradicting information under time pressure, limiting decision making in the short term and learning in the long term, as was also found in two related audit-specific studies (Choo, 1995; Glover, 1997). Consequently, we hypothesize the following:

*Hypothesis* 1d: Professionals will report less error learning when they experience more (rather than less) time pressure.

### 2.2. The moderating role of EMC for error learning

We explore whether the negative relationships between the four work conditions and error learning is moderated by auditors’ perceptions of the *error management climate* (EMC). In their landmark paper, van Dyck et al. (2005) distinguish between two facets of EMC; an *open EMC* is characterized by organizations or management showing a high tolerance for making errors, as long as learning occurs and errors are not repeated. At the other extreme, in a *blame EMC*, making errors is considered unacceptable and therefore should be penalized to prevent reoccurrence. We aim to understand whether perceptions of an open EMC can mitigate the negative consequences of the four work conditions for error learning, while perceptions of a blame EMC may exacerbate the negative relationships predicted in Hypothesis 1a–d. This argument builds on Salancik and Pfeffer’s (1978) social information-processing theory, which posits that individuals interpret their work environment through their own personal lens based on past experiences, socialization, and personal values, and that this personal interpretation, rather than the work environment itself, drives an individual’s attitudes and behaviors within the work context. For non-error-specific learning, this notion is empirically shown by, for example, Eldor and Harpaz (2016), Park and Rothwell (2009), and Mikkelsen et al. (1998). Consequently, we focus on individual perceptions of error management values, beliefs, and behaviors within audit firms in relation to the four work conditions and individual-level error learning.

#### 2.2.1. Open EMC perceptions as moderator

An open EMC is driven by management that actively encourages organizational members to learn from their errors by emphasizing the omnipresence of errors, encouraging them to analyze error causes to develop strategies for preventing the same errors from re-occurring in the future, and shielding individuals who make errors from being punished (van Dyck et al., 2005; Keith and Frese, 2011). Prior audit research shows that an open EMC increases auditors’ willingness to share errors after discovering them (Gronewold et al., 2013; Gold et al., 2014), and that it affects the degree to which auditors feel responsible for acting on errors (Gronewold and Donle, 2011). To date, audit research on EMC focuses on error reporting (e.g., Stefaniak and Robertson, 2010; Gronewold et al., 2013); research on actual error learning in this context is rare (Gold et al., 2022; Smeets et al., 2022), as are studies that explore the interaction of perceived EMC with other factors (van Dyck et al., 2005; Zhao and Olivera, 2006; Gronewold and Donle, 2011). Consequently, we also build on extant findings in other contexts and on related concepts to develop our next hypotheses.

First, regarding small error consequences, Stefaniak and Robertson (2010) showed that lower-ranking auditors are more likely to report errors when their direct supervisors did not punish errors in the past. They conclude that supervisor behavior communicates acceptable behavior within a group, so that learning from errors—even when the error consequences are small—is increased by open EMC perceptions. As a result, we predict that the negative effect of small error consequences on error learning is mitigated by open EMC perceptions. Second, while prior research acknowledges that error type matters for the effectiveness of error management (e.g., Zhao and Olivera, 2006), we were unable to identify prior research on its interactions with EMC. However, an open EMC emphasizes learning potential across all error types (e.g., van Dyck et al., 2005), which should mitigate the difference between routine and non-routine errors for learning. Third, concerning strong negative emotions, research suggests that subordinate emotions are closely related to leader expectations and attitudes (i.e., Edmondson, 2004; Gronewold and Donle, 2011; Zhao, 2011). Both Smeets et al. (2022) and Grohnert et al. (2019) found that beginning auditors engage in more error learning when they perceive that they work in a supportive climate for learning from errors, while also experiencing less strain. Consequently, we expect open EMC perceptions to mitigate the negative relationship between negative emotions and error learning. Finally, studies on error learning frequently mention time pressure as a characteristic of the environment in which errors are made, but do not explicitly study its impact on error learning or how it interacts with climate (i.e., Zhao and Olivera, 2006). In one recent study, Zhao et al. (2022) found that professionals working in an open EMC reported more learning from errors, even when faced with work stressors that include time pressure. We therefore expect auditors’ open EMC perceptions will alleviate the negative relationship between high time pressure and error learning. This leads to our first interaction hypothesis:

*Hypothesis* 2: Professionals’ perceptions of working in an open EMC mitigate the negative relationship between error learning and (a) small rather than large error consequences, (b) routine rather than non-routine errors, (c) stronger rather than weaker negative emotions, and (d) more rather than less time pressure.

#### 2.2.2. Blame EMC perceptions as moderator

**Figure 1:**
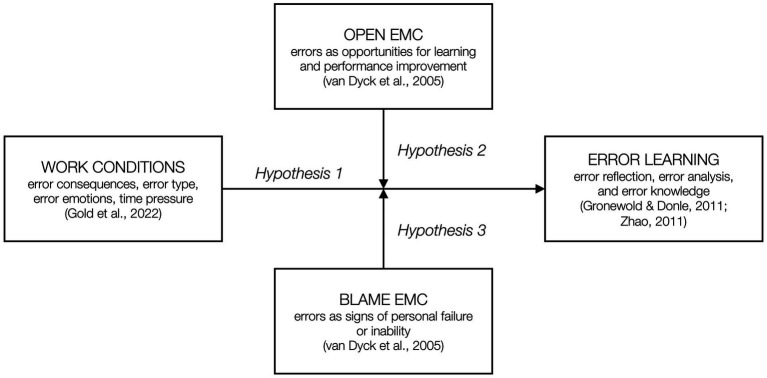
Conceptual model.

In contrast to an open EMC, in a blame EMC, upper management shares an attitude of ‘getting things right the first time’, without acknowledging that eradicating all errors is impossible. Initiatives to learn from errors are, at best, lip service, and organizational members are led to believe that making errors leads to formal and informal sanctions, such as lower performance evaluations (van Dyck et al., 2005; Frese and Keith, 2015). We expect that such a blame EMC perception will exacerbate the negative relationships between the four work conditions and error learning predicted in Hypothesis 1. Overall, we found fewer studies focusing specifically on a blame EMC (and related concepts), compared to research on an open EMC. The studies by Fruhen and Keith (2014) and Matthews et al. (2022) are two exceptions, showing that in a blame EMC, errors are actually more likely to occur, although professionals are not more likely to manage these errors, for example, by learning. Further, working in a blame EMC is associated with negative emotions of fear and stress resulting from blame and punishment (Gorini et al., 2012; Matthews et al., 2022), suggesting a greater likelihood for strong negative emotions to adversely affect error learning. We were unable to identify studies that explicitly link perceptions of a blame EMC to error type and time pressure with regard to (error) learning; however, the theoretical logic maintains that the negative relationships predicted in Hypotheses 1b and 1d are likely to be exacerbated when professionals perceive the EMC to be one of blame. Based on the established outcomes associated with a blame EMC and related concepts in prior research, we formulate our second and final moderation hypothesis. All hypotheses are illustrated in Figure 1, our conceptual model.

*Hypothesis* 3: Professionals’ perceptions of working in a blame EMC exacerbate the negative relationship between error learning and (a) small rather than large error consequences, (b) routine rather than non-routine errors, (c) stronger rather than weaker negative emotions, and (d) more rather than less time pressure.

## 3. Materials and methods

### 3.1. Setting and sample

We contacted 247 practicing auditors across hierarchical ranks working for two different audit firms in Netherlands. In one firm, participants completed an experiential survey as part of firm-internal in-person training sessions, in the presence of at least one of the authors. Authors present during the study were unknown to the participants and provided support in case of technical difficulties, along with a briefing and debriefing. These trainings were endorsed by top management, so that mandatory attendance of participants limited self-selection bias. The other firm recruited participants as part of internal training sessions for qualifying auditors, meaning in the first 3 years of practice, and provided dedicated time for completing our instrument. Screening of the responses revealed that 98 responses were incomplete (91 participants abandoned the study after reading the instructions; seven participants indicated that they did not have an error to report before abandoning the instrument), a further eight observations were excluded as the entire instrument was completed in an unrealistically short timeframe, less than 5 minutes. Our final sample therefore includes 141 usable records (57% of the total response). Auditors in this sample are between 21 and 56 years of age (*M* = 29.87, SD = 7.46), and 30% are publicly registered accountants in Netherlands. Our sample includes 44 staff auditors, the lowest rank, followed by 34 senior staff auditors, 27 managers, 16 senior managers, and eight directors/partners, the last being the highest rank (12 missing values), and includes 67% male participants, in line with the pyramid structure and common gender balance in audit firms (EY, 2018; KPMG, 2018; PwC, 2018). Moreover, 93% work in an audit function, 6% work in audit-adjacent roles, such as advisory (two missing values); 92% work for a large audit firm, while 7% work for a smaller firm (one missing value).

### 3.2. Materials and measures

The starting point of our experiential questionnaire was developed in close collaboration with an experienced audit partner, asking participants to recall and describe a specific error, which was the focus for the remainder of the questionnaire. After assuring participants of the anonymity and confidentiality of all information provided, the experiential questionnaire started with the following definition of an error: “Any unintentional action or omission by you that caused consequences for audit planning, procedures or other goals during your or your peers’ audit work.” We acknowledge that this definition departs somewhat from common definitions in the error literature, where errors are explicitly separated from their consequences (e.g., van Dyck, 2009; Klamar et al., 2022). Rather than presenting an academic definition of errors, the description we provided to survey respondents was somewhat narrower than definitions commonly used in the literature (Rasmussen, 1986; Reason, 1990; Sitkin, 1992; Zhao and Olivera, 2006), as we limited the requested respondent recall to errors that were in fact followed by consequences. There were two reasons for this instruction, which was developed together with a practicing auditor. First, when auditors are confronted with the term ‘error’ they immediately associate it with errors made by their client (i.e., financial statement errors). We intentionally aimed to direct their attention to errors made by themselves that would potentially affect not only the client, but also their own or their peers’ work. As a result, we emphasized the notion of consequences in the survey definition. Second, since one of our key interests lies in the effect of error consequences on error learning, we aimed to direct respondents’ attention to errors that would carry variability in consequences. While some of the errors we target may indeed be classified as failure in the literature, we refrained from using this value-laden label in our survey. Finally, we recognize that the definition might bias responses away from errors with zero consequences; on the other hand, we expected that recall of such errors might be relatively minor. As will be shown, we find substantial variance in the extent to which reported errors carry consequences (including zero-consequence errors), as intended.

We then asked participants to describe an error that fit the provided error definition. Following the general prompt, participants answered a range of closed-ended, Likert-type, and open-ended questions that measured the relation between the described error and the inhibiting factors, perceived EMC, and learning from the error. In developing the open-ended questions (see Appendix), we avoided leading questions to increase accuracy and the participant’s recall and reporting (i.e., Downey and Bedard, 2019). The questionnaire was presented in English using original scale items. In a final step, participants provided demographic information.

#### 3.2.1. Error learning

In line with the definition of learning from errors by Zhao (2011), we measured *error learning* using Gronewold and Donle’s (2011) audit-specific adaptation of Rybowiak et al.’s (1999) scales for error reflection, error analysis, and error knowledge. To suit the setting of the error-focused experiential survey, we adapted the instrument in two ways. First, the original statements (e.g., “I often think about how an error might have been avoided”) were reformulated into past tense statements, and participants were instructed to provide answers based on their chosen error, rather than on general practices within their firm. Participants responded to 11 items (measured on a scale ranging from 1 “strongly disagree” to 5 “strongly agree”). The first sub-scale, error reflection, captures participants’ behavior in purposefully reflecting after discovering errors (example item: “After the error, I thoroughly thought thoroughly about how to correct it”). The second sub-scale, error analysis, reflects whether participants analyzed the cause of their errors (example item: “Because I made a mistake, I analyzed it thoroughly.”). Finally, the sub-scale for error knowledge indicates whether participants were able to create insights that help to improve future behavior or decision making (example item: “I learned a lot from my error for mastering my work.”). We performed principal component analysis (PCA) with direct oblimin rotation to explore our adapted scale. We accepted a one-component solution after the removal of two items, resulting in a reliable overall scale (Cronbach’s alpha = 0.799)^1^. We therefore calculate the mean value of the remaining nine items as our measure for the dependent variable *error learning*.

#### 3.2.2. Work conditions that may inhibit error learning

Following the interview study by Gold et al. (2022), we measure four factors that may inhibit learning from errors: error consequence, error type, error emotions, and time pressure. First, to measure *error consequence*, we inductively coded participants’ descriptions of the error made and the consequences reported by distinguishing whether errors resulted in adjustments to procedures, additional work for colleagues, or an additional information request from the client. These criteria were developed with an experienced audit partner and applied by the second author and an independent coder with audit experience as 0 = no error consequences (*n =* 41), 1 = small error consequences (*n =* 57), and 2 = large error consequences (*n =* 25; 20 error consequences could not be categorized with the information given). Interrater reliability was high (Cohen’s kappa = 0.80, *p* < 0.01).

Second, *error type* was coded as either routine or non-routine by the third author and an independent coder with audit experience, in line with the conceptualizations by Sutcliffe and Rugg (1998) and Zhang et al. (2019), both based on Rasmussen (1986). During the coding process, both raters initially distinguished between skills-, rule-, and knowledge-based errors; 17 errors could not be coded. At this stage, interrater reliability was low (Cohen’s kappa = 0.38, *p* < 0.01), due to different perceptions about the nature of a skills-based error as resulting either from the person’s perception or from the nature of the task performed. After calibration between coders, skills-based errors were coded from the perspective of the auditor making the error (1 = routine error, *n* = 42), and rule-and knowledge-based error were grouped together (2 = non-routine error, *n* = 84), which resulted in high interrater reliability (Cohen’s kappa = 0.85, *p <* 0.01).

Third, *negative emotions* are captured using the circumplex model in line with Rausch et al. (2017), with eight emotional categories, four positive and four negative. We asked participants to select at least three of the eight emotional categories and to indicate how intensely they experienced their chosen emotional states on a 3-point scale (“1” a little, “2” somewhat, “3” intensely). Most (79.7%) of participants indicated that they felt unhappy/gloomy/sad (mean intensity = 2.04, SD = 0.693, *n* = 115), followed by 72% who were irritated/annoyed/angry (mean intensity = 1.97, SD = 0.760, *n* = 103), and 67.8% who experienced nervousness/worry/fear (mean intensity = 1.96). The negative emotions of boredom/dullness/disinterest were only selected by 4.9%. At the same time, positive emotions were only reported by 11.9–30.1% of the auditors, with a mean intensity of 1.96. These positive emotions include (1) motivated/delighted/curious (selected by 30.1%), (2) confident/happy/glad (selected by 11.9%), (3) contented/accepted/proud (selected by 12.6%), and (4) calm/even-tempered/daydreaming (selected by 21.7%). Prior studies including multiple emotions at the same time often include them as separate variables (i.e., Reio and Callahan, 2004), or use a mean score across different emotions to indicate the presence of certain emotions (i.e., Scott and Sutton, 2009). Following Hypothesis 1c and taking a quantitative approach, we operationalize *error emotions* as the sum score of the emotional intensity of the dominant three negative emotions selected by participants, focusing on intensity of experience of negative emotions.

Finally, we captured *time pressure* in a manner as closely as possible related to auditors’ actual time perceptions. Auditors in practice are given a budget of time paid for by the client to complete a certain task, and auditors refer to these budgets when discussing time pressure (Kelley and Margheim, 1990; Gold et al., 2022). We therefore asked what percentage of additional time participants would have liked to use for managing the error compared to the available time at that moment (measured on a 5-point scale and ranging from “between 0 and 20%” to “between 80 and 100%). This measure anchors all participants on a fixed entity, eliciting a relative measure that is more easily compared across participants, given individual differences in perception of time pressure (e.g., Margheim et al., 2005). *Time pressure* is measured as participants’ indication of their perceived additional time needed (69 reported 0–20% extra time needed, 19 reported 21–40%, and 9 wanted more than 41% extra time).

#### 3.2.3. Error management climate

We captured two facets of the participants’ firm’s perceived error management climate using van Dyck et al.’s (2005) validated instrument. *Perceived open EMC* is measured through 17 items on a five-point scale, including “After an error, people think through how to correct it” and “Our errors point us at what we can improve.” *Perceived blame EMC* is measured by 11 items, including “In this organization, people feel stressed when making mistakes” and “There are advantages in covering up one’s errors.” Due to a formatting error that occurred when creating the online survey, one item from each scale was accidentally omitted (“After making a mistake, people try to analyze what caused it” and “During their work, people are often concerned that errors might occur”), resulting in 16 and 10 items, respectively. PCA analysis with direct oblimin rotation reveals a two-component solution in line with van Dyck et al.’s original scales, which is also reflected in the reliability scores for both scales, open and blame EMC (Cronbach’s alpha = 0.848 and 0.802, respectively). *Error management climate* is therefore measured through participants’ mean perceived values for both the open and the blame EMC items at the individual level.

#### 3.2.4. Control variables

Given prior findings that professionals can experience error-related strain and vulnerability differently depending on a range of characteristics (Metcalfe, 2017; Grohnert et al., 2019), we include five covariates in our analyses to represent findings from prior research as well as to account for the current research setting. In line with the conceptual model of individual error learning by Tulis et al. (2016), we include three covariates that represent our sample characteristics: *gender*, *certification* status, and *rank*, as described in the sample setting above. Additionally, women tend to be in the minority within auditing, especially at higher ranks, which has been associated with increased vulnerability in prior studies (Hardies et al., 2011). In line with this notion, Grohnert et al. (2017) found that female auditors were more likely to cover up errors, an action that inhibits learning (e.g., van Dyck et al., 2005). Regarding experience and rank, prior studies found that professionals with more experience tend to report more learning from errors as well, which may be related to their status in the organization and/or to the ability to make better sense of errors with more prior knowledge (Carmeli and Gittell, 2009; Smeets et al., 2022). Methodologically, we account for memory effects by including the number of months that have passed between the error occurrence and completing the experiential questionnaire (*timing*), as reported by participants (Mahajan, 2010; Rausch et al., 2017). Finally, we include *firm type* as a covariate to take into account variance in EMC across employers (van Dyck et al., 2005; Vera and Crossan, 2016).

### 3.3. Analysis strategy

After reporting descriptive statistics and correlations, we explore our hypotheses through conditional process modelling using Hayes’ (2018) PROCESS macro for SPSS, and specifically model 2, the moderation model, given our sample size. Conditional process models estimate the conditional interaction effects and generate bias-corrected 95% confidence intervals (CI) for the interaction effects at various values of the moderator variable (*M* − 1SD, *M*, *M* + 1SD, labeled as low, medium and high, respectively; Aiken and West, 1991; Hayes, 2018). Due to the fact that many participants in our sample work for the same organization, we made use of robust standard errors to account for a potential violation of the independence assumption of OLS regression (Hayes and Cai, 2007). In a preliminary step, we used Harman’s single factor test to check for common method bias; with 15.5% of variance explained, common method bias is unlikely to affect our results.

## 4. Results

### 4.1. Descriptive statistics and correlations

Table 1 reports the means, standard deviations, and correlations for all study variables. In line with the categorical descriptive statistics reported under 3.2.2 we find that overall, auditors reported errors with mostly small consequences that are mostly non-routine, they experienced medium-strength negative emotions, and required around 20% additional time to adequately manage the error. At the same time, auditors reported working in an EMC that is more open (*M* = 3.91 out of 5, SD = 0.46) than blame-oriented (*M* = 2.87 out of 5, SD = 0.64), along with medium to high error learning (*M =* 3.51 out of 5, SD = 0.58). These distributions inform our findings. Examining the correlations, we find that error learning correlates positively with error consequences (*r* = 0.22, *p* < 0.05) and error emotions (*r* = 0.38, *p* < 0.001), but does not correlate significantly with error type and time pressure. In turn, an open EMC correlates negatively with a blame EMC (*r* = −0.32, *p* < 0.001) and with time pressure (*r* = −0.30, *p* < 0.01), and a blame EMC correlates positively with error emotions (*r* = 0.33, *p* < 0.05) and time pressure (*r* = 0.19, *p* < 0.10). Regarding our covariates, female relative to male auditors reflected on errors that took place further in the past (*r =* 0.23, *p <* 0.01), with larger consequences (*r =* 0.25, *p <* 0.01) and more time pressure (*r =* 0.26, *p <* 0.05). Auditors of higher relative to lower ranks also reported errors that took place further in the past (*r =* 0.26, *p <* 0.01), were less likely to work at smaller audit firms (*r* = −0.38, *p* < 0.001), and experienced a more open EMC (*r =* 0.17, *p <* 0.05) and more error learning (*r* = 0.33, *p* < 0.001).

**Table 1:** Means, standard deviations, and correlations among variables.

Variable	*M*	SD	(1)	(2)	(3)	(4)	(5)	(6)	(7)	(8)	(9)	(10)	(11)
(1) Gender	1.33	0.47	1										
(2) Certification	1.70	0.46	0.02	1									
(3) Rank	2.29	1.21	0.02	0.79*^**^	1								
(4) Timing	9.66	14.37	0.23^**^	0.27^***^	0.26^**^	1							
(5) Firm Type	1.15	0.36	0.02	−0.27^**^	−0.38^***^	−0.13	1						
(6) Consequences	1.13	0.71	0.25^**^	0.04	0.02	0.17^+^	0.07	1					
(7) Type	1.71	0.43	0.06	0.20^*^	0.11	−0.10	0.03	−0.07	1				
(8) Emotions	5.93	1.25	0.13	0.07	0.12	−0.03	−0.13	0.15	0.09	1			
(9) Time Pressure	1.40	0.72	0.26^*^	0.02	0.06	0.03	0.11	−0.01	−0.18^+^	0.113	1		
(10) Open EMC	3.91	0.46	−0.13	0.15	0.17^*^	−0.07	0.03	0.13	0.05	−0.12	−0.30^**^	1	
(11) Blame EMC	2.87	0.64	0.11	0.09	0.11	0.08	−0.17^*^	0.04	−0.11	0.33^*^	0.19^+^	−0.32^***^	1
(12) Error Learning	3.51	0.58	0.08	0.30^***^	0.33^***^	0.09	−0.03	0.22^*^	0.02	0.38^***^	0.08	0.32^***^	0.06

*N* = 141 individuals. For gender, 1 = male, 2 = female, 3 = prefer not to say. For role, 1 = audit, 2 = other. For certification, 0 = no, 1 = yes. For rank, 1 = staff, 2 = senior, 3 = manager, 4 = senior, 5 = director/partner. For timing, scores represent months between error commission and completing the instrument. For firm type, 1 = large firm, 2 = small firm. For consequence, score is between 1 and 3, no to large error consequence. For type, 1 = routine error, 2 = non-routine error. For emotions, score is how intensely participants felt nervous/worried/afraid on a scale from 0 = not at all to 3 = intensely (Rausch et al., 2017). For time pressure, score is between 1 and 5, from 0 to 100% extra time required to address error. For open and blame EMC, score is between 1–5, based on van Dyck et al.’s EMC and EAC instruments (2005). For error learning, score is between 1–5, based on Gronewold and Donle’s instrument (2011). Significance of correlations is indicated as ^+^ = *p* < 0.10, ^*^p < 0.05, ^**^p < 0.01, ^***^p < 0.001 (2-tailed).

### 4.2. Hypothesis testing

#### 4.2.1. Error consequences, open/blame EMC, and error learning (H1a, H2a, H3a)

**Figure 2:**
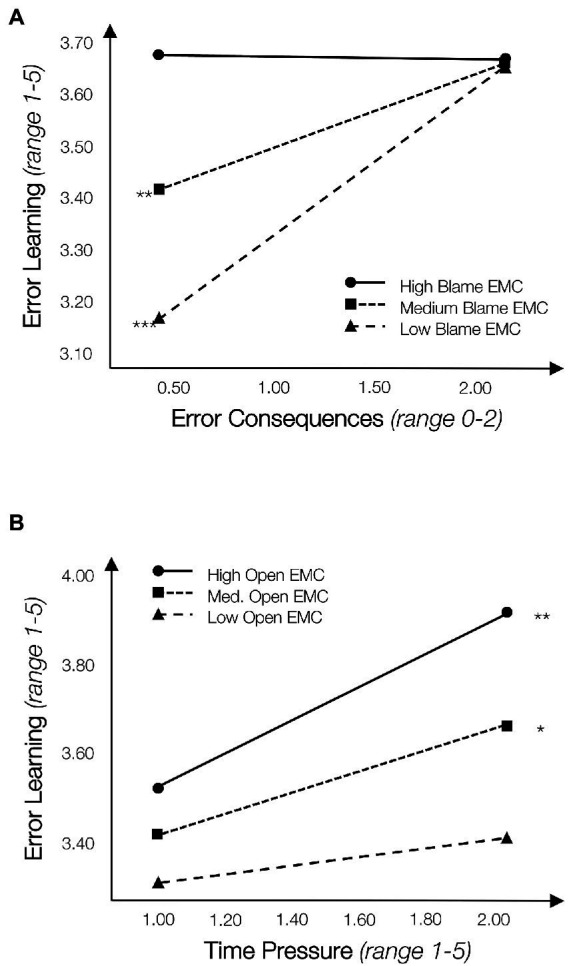
Moderating role of open and blame error management climate (EMC) in the relationship between error consequences, time pressure, and error learning. **(A)** Consequences × blame EMC. **(B)** Time pressure × open EMC caption: *N* = 141; conditional effects are illustrated using the pick-a-point method, where continuous moderators are binned at *M* − 1SD, Mean, *M* + 1SD (Aiken and West, 1991; Hayes, 2018). Interactions are calculated separately for open EMC and blame EMC. We report unstandardized coefficients and *p*-values. Significance is indicated as + = *p* < 0.10, ^*^p < 0.05, ^**^p < 0.01, ^***^p < 0.001 (2-tailed).

Table 2 reports moderation analyses relating our covariates, the four work conditions along with the measures for open/blame EMC, and their interactions to error learning. Model 1 in Table 2 reports the findings for Hypotheses 1a, 2a and 3a, relating error consequences, open and blame EMC to error learning. Overall, this model is significant (*F*(10,109) = 5.21, *p* < 0.001) explaining 32% of the variance in error learning. In line with Hypothesis 1a, we find that error consequences relate positively to error learning, so that less learning takes place from errors with smaller consequences (*B* = 1.80, *p* < 0.05; SE = 0.81). Contrary to Hypothesis 2a, we do not find a significant interaction for an open EMC (*B* = −0.20, *p* > 0.10; SE = 0.15). At the same time, with respect to Hypothesis 3a, we find a significant interaction for a blame EMC (*B* = −0.30, *p* < 0.05; SE = 0.14, Δ*R*^2^ Blame = 0.05, *p* < 0.05). Figure 2 Panel A illustrates this interaction effect through three conditional effects: more error learning takes place from errors with larger compared to smaller consequences when auditors perceive themselves to work in a low (*M* − 1SD; *B*_low_ = 0.349, *p* < 0.001) and medium (*M*; *B*_medium_ = 0.172, *p* < 0.01) blame EMC. However, in a high blame EMC, auditors reported equal and high error learning regardless of error consequences (*M + 1*SD; *B*_high_ *=* −0.005, *p* > 0.10; see Figure 2, Panel A). This conditional effect is contrary to Hypothesis 3a: while we expected that perceptions of a blame EMC would further exacerbate the negative effect of small error consequences on learning, we find instead that blame EMC perceptions mitigate this relationship.

**Table 2:** OLS Moderation Analysis.

	Model 1		Model 2		Model 3		Model 4	
	*B*	SE	*B*	SE	*B*	SE	*B*	SE
*Variable*								
Gender	0.01	0.12	0.08	0.11	0.08	0.21	0.05	0.14
Certification	0.06	0.14	0.16	0.16	0.21	0.30	0.22	0.18
Rank	0.122^**^	0.05	0.09	0.05	0.13	0.12	0.04	0.08
Timing	0.00	0.01	0.00	0.01	0.00	0.01	0.00	0.00
Firm Type	0.12	0.17	0.13	0.16	0.00	0.01	0.16	0.16
Consequence	1.80^*^	0.81						
Type			−1.83	2.17				
Emotions					−0.63	1.20		
Time Pressure							−1.68	1.20
Open EMC	0.50^**^	0.18	−0.15	0.39	1.14	1.42	−0.16	0.33
Blame EMC	0.58^***^	0.19	−0.27	0.22	0.64	1.15	−0.01	0.25
Consequences × open	−0.20	0.15						
Consequences × blame	−0.30^*^	0.14						
Type × open			0.32	0.22				
Type × blame			0.19	0.18				
Emotions × open					0.25	0.23		
Emotions × blame					−0.07	0.19		
Time × open							0.41^+^	0.24
Time × blame							0.11	0.17
*Model*								
*F*-statistic	5.21^***^		3.71^***^		4.04^***^		2.81^***^	
*R* ^2^	0.32		0.22		0.48		0.23	
Δ*R*^2^ open	0.01		0.01		0.05^*^		0.05	
Δ*R*^2^ blame	0.05^*^		0.01		0.01		0.01	

*N* = 141. Table reports unstandardized regression coefficients and robust standard errors (Davidson-MacKinnon; Hayes and Cai, 2007). Significance is indicated as ^+^*p* < 0.10, ^*^p < 0.05, ^**^p < 0.01, and ^***^p < 0.001 (2-tailed).

#### 4.2.2. Error type, open/blame EMC, and error learning (H1b, H2b, H3b)

Considering Hypotheses 1b, 2b, and 3b focusing on error type in interaction with open/blame EMC for error learning, we report our findings in Table 2, Model 2. This model is significant (*F*(10,112) = 3.71, *p* < 0.001), explaining 22% of the variance. Yet, contrary to Hypotheses 1a, 2b, and 3b, we find neither significant direct relationships nor interactions. We therefore do not find support for the hypothesis that routine errors can impede error learning, nor for the expectation that this relationship is moderated by an open or a blame EMC.

#### 4.2.3. Negative emotions, open/blame EMC, and error learning (H1c, H2c, H3c)

Table 2, Model 3 reports the findings for Hypotheses 1c, 2c, and 3c, exploring the links between negative emotions, open/blame EMC, and error learning. This model is significant (*F*(10,44) = 4.04, *p* < 0.001), explaining 48% of the variance. Contrary to Hypothesis 1c, we do not find a significant direct effect of negative emotions, and we find no significant interactions with perceptions of an open or a blame EMC in line with Hypothesis 2c and 3c. Yet, the model explains a high percentage of variance, and adding the interaction term between negative emotions and an open EMC significantly increased the variance explained of the model (Δ*R*^2^ Open = 0.05, *p* < 0.05). We therefore performed additional post-hoc analyses to explore Hypotheses 1c, 2c, and 2c further. Please refer to section 4.3.1 below.

#### 4.2.4. Time pressure, open/blame EMC, and error learning (H1d, H2d, H3d)

Finally, Table 2, Model 4 reports our findings on Hypotheses 1d, 2d, and 3d with respect to time pressure, open/blame EMC and error learning. Overall, the model is significant (*F*(10,83) = 2.81, *p* < 0.001), explaining 23% of the variance in error learning. Contrary to Hypothesis 1d, we do not find a significant direct relationship between time pressure and error learning. Regarding Hypothesis 2d, we find a significant interaction between time pressure and an open EMC (*B* = 0.41, *p* < 0.10; SE = 0.24). Figure 2, Panel B illustrates this interaction through three conditional effects, showing that more error learning was reported with more time pressure when participants experienced high (*M +* 1SD; *B*_high_ = 0.380, *p* < 0.01) and medium open EMC (*M*; *B*_medium_ = 0.234, *p* < 0.05). At the same time, reported error learning under high and medium open EMC is higher for auditors experiencing higher time pressure compared to lower time pressure. Open EMC perceptions therefore did not mitigate the negative relationship between time pressure and error learning, but rather fostered learning from errors specifically under conditions of high time pressure. Finally, we do not find a significant interaction with blame EMC. Hence, we find no support for Hypothesis 3d.

### 4.3. Additional analyses

Following up on our limited findings regarding negative emotions and time pressure, we performed additional *post-hoc* analyses for the relationships posited in Hypotheses 1c/2c/3c and 1d.

#### 4.3.1. *Post-hoc* analysis of the relationship between negative emotions, open/blame EMC, and error learning

We found that Model 3 in Table 2 explained a high percentage of the variance in error learning (48%) in the absence of significant coefficients. At the same time, the coefficient representing the interaction between negative emotions and open EMC was insignificant, while it contributed significantly to the model’s variance explained. To explore these findings further, we performed two *post-hoc* analyses. First, to test whether our non-significant results are driven by the correlations between error learning, open EMC, and negative emotions (see Table 1), we orthogonalized these three variables and reran Model 3 (Little et al., 2006). Orthogonalizing variables means setting the correlations between variables to zero, so that in an interaction model, entering the interaction term does not affect the partial regression coefficients of the main effects. This approach has been associated with more stable regression findings with correlated variables, especially in interaction models (Little et al., 2006). Table 3 reports the original OLS model (Model 1), alongside the orthogonalized one (Model 2). Model 2 is just as significant as Model 1 (*F*(10,44) = 4.61, *p* < 0.001), explaining 27% of the variance. We find significant coefficients for negative emotions (*B* = −0.13, *p* < 0.05, SE = 0.05). In line with Hypothesis 1c, we find participants reported less error learning with more intense negative emotions. At the same time, the interaction terms are insignificant, just as in Model 1.

**Table 3:** Additional analyses of the emotions—error learning relationship.

	Model 1		Model 2		Model 3		Model 4	
	*B*	SE	*B*	SE	*B*	SE	*B*	SE
*Variable*								
Gender	0.08	0.21	0.08	0.10	0.10	0.19	0.06	0.22
Certification	0.21	0.30	−0.14	0.13	−0.25	0.28	−0.30	0.35
Rank	0.13	0.12	0.10^+^	0.06	0.14	0.10	0.12	0.12
Timing	0.00	0.01	0.00	0.00	0.00	0.01	0.00	0.01
Firm type	0.00	0.01	0.20	0.16	0.03	0.27	0.00	0.26
Emotions	−0.63	1.20	−0.13^**^	0.05	−1.11	0.79	0.76^+^	0.39
Open EMC	−1.14	1.42	0.16^***^	0.05	−1.81	1.18		
Blame EMC	0.64	1.15	0.06	0.05			1.33^+^	0.73
Emotions × open	0.25	0.23	0.05	0.04	0.34^+^	0.20		
Emotions × blame	−0.07	0.19	0.00	0.04			−0.20^+^	0.12
*Model*								
*F*-statistic	4.04^***^		4.61^***^		2.29^*^		1.68	
*R* ^2^	0.48		0.27		0.43		0.34	
Δ*R*^2^ open	0.05^*^		0.01		0.12^+^			
Δ*R*^2^ blame	0.01		0.00				0.08^+^	

*N* = 141. Table reports unstandardized regression coefficients and robust standard errors (Davidson-MacKinnon; Hayes and Cai, 2007) for Model 1, 3, and 4. Model 2 reports the results for orthogonalized error emotions, open/blame EMC, and error learning [in line with Little et al., 2006]. Significance is indicated as ^+^*p* < 0.10, ^*^p < 0.05, ^**^p < 0.01, and ^***^p < 0.001 (2-tailed).

**Figure 3:**
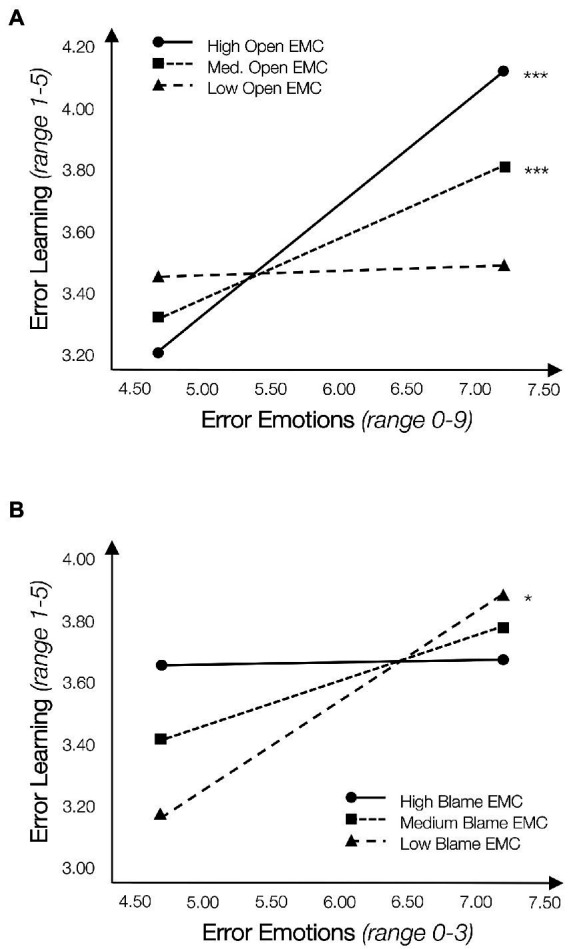
Additional analyses negative emotions and error learning. **(A)** Emotions × open EMC. **(B)** Emotions × blame EMC. *N* = 141; conditional effects are illustrated using the pick-a-point method, where continuous moderators are binned at *M* − 1SD, Mean, *M* + 1SD (Aiken and West, 1991; Hayes, 2018). Interactions are calculated separately for open EMC and blame EMC. We report unstandardized coefficients and *p*-values. Significance is indicated as + = *p* < 0.10, ^*^p < 0.05, ^**^p < 0.01, ^***^p < 0.001 (2-tailed).

Yet, given the significant amount of variance explained that remains even after orthogonalizing our variables, we ran the analysis separately for an open and a blame EMC as moderators, respectively. By splitting the analysis, we identify distinct effects of both EMC facets that appear to overlap when included at the same time (see Table 3, Models 3 and 4). First, we consider the interaction of negative emotions with open EMC with regard to error learning. This model is significant (*F*(8,85) = 2.29, *p* < 0.05, *R*^2^ = 0.43) and we find a significant interaction effect (*B* = 0.34, *p* < 0.10; SE = 0.20, Δ*R*^2^ Open = 0.12, *p* < 0.10; see Table 3, Model 3). Figure 3, Panel A graphically represents this interaction through three conditional effects: more error learning occurred with more intense negative emotions the more open the EMC was perceived to be. Regarding Hypothesis 2c, we find significant slopes for high (*M* + 1SD, *B*_high_ *=* 0.356, *p* < 0.001) and medium (*M*; *B*_medium_ *=* 0.187, *p* < 0.001) open EMC scores, suggesting that a high or medium open EMC increases reported learning from errors even given strong negative emotions. The same analyses performed for blame EMC perceptions reveal a significant interaction (*B* = −0.20, *p* < 0.10; SE = 0.12, Δ*R*^2^ Blame = 0.08, *p* < 0.10; see Table 3, Model 4). This model explains significant variance, but fit is suboptimal, with a significant percentage of variance explained (*F*(8,85) = 1.68, *p* > 0.10; *R*^2^ = 0.34). Exploring this interaction visually (Figure 3, Panel B), we find a significant slope for low blame EMC (*M*–1SD; *B*_low_ = 0.279, *p* < 0.05), showing more learning takes place with more intense negative emotions; at the same time, auditors report the same amount of error learning from weaker and stronger negative emotions with medium and high blame EMC, which is contrary to Hypothesis 3c.

#### 4.3.2. *Post-hoc* analysis of the relationship between time pressure and error learning

**Figure 4:**
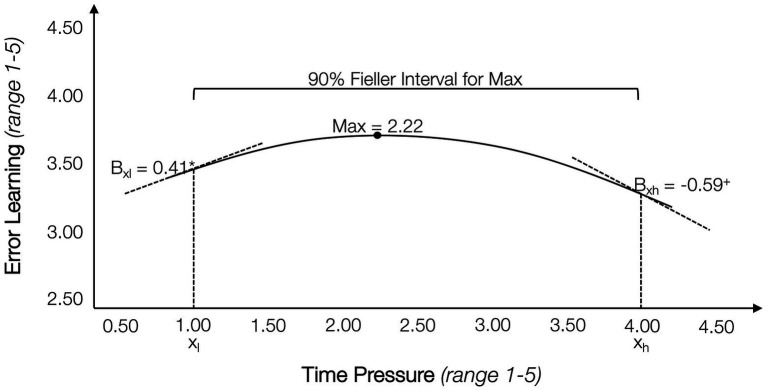
Non-linear relationship between time pressure and error learning. *N* = 141; non-linear relationship tested using Lind and Mehlum’s (2010) approach of calculating a 90% Fieller interval around the apex of the function, comparing sign and strength of the slopes at the lower and upper boundaries with a *t*-test.

Following up on the insignificant main effect of time pressure on error learning (Hypothesis 1d), we conducted a *post-hoc* analysis to explore a potential non-linear relationship. A series of extant studies on time pressure have found that medium levels of time pressure can be positively associated with a range of outcomes, such as creativity, learning, or performance, for example, by creating a sense of urgency or motivating action (e.g., Spilker, 1995; Baer and Oldham, 2006; Prem et al., 2017; Ryari et al., 2021). At the same time, higher levels of time pressure can impede these outcomes, for example, by shifting priorities, or by occupying working memory through creating stress. We therefore explore whether the relationship between time pressure and error learning in our sample is also characterized by an inverted-U shaped function. Lind and Mehlum (2010) propose a methodology for testing non-linear relationships not through regression, but by comparing slopes on either side of the apex. With this approach, two points are selected on the curve, representing a 90% Fieller interval of the apex, comparing whether the slope of the lower point is positive and significantly different from the negative slope of the upper point. Using the ‘utest’ command in Stata yields a *t*-value of 1.51, with a one-sided value of *p* of 0.03. Figure 4 shows that the relationship between time pressure and error learning is indeed characterized by an inverted-U shape, suggesting that a low level of time pressure can be beneficial for reported learning from errors while higher levels of time pressure have an adverse effect, as predicted in H1d.

## 5. Discussion

In this study, we explored four work conditions that may impede learning from errors during professionals’ work. In the context of financial auditing, we explored whether professionals’ perceived EMC moderates the negative relationship of small error consequences, routine-type errors, strong negative emotions, and high time pressure with error learning. Our analyses of experiential questionnaire data from 141 practicing auditors resulted in three key findings. First, perceptions of an open EMC positively moderate the relationship between strong negative emotions, high time pressure, and error learning. These results contribute to extant research showing a positive direct relationship between open EMC and error learning (i.e., Grohnert et al., 2019; Smeets et al., 2022), and to studies relating emotions (Ilgen and Davis, 2000; Heimbeck et al., 2003; Zhao et al., 2019) and time pressure (Kruglanski et al., 1993; Zhao and Olivera, 2006; Homsma et al., 2009) to error learning. Second, we also found significant moderation by blame EMC perceptions—albeit not always in line with our predictions. Regarding negative emotions, auditors engaged in more learning from errors accompanied by strong negative emotions when they perceived themselves to work in an EMC with low blame characteristics. In the absence of prior research on this relationship, we find that an absence of blame values and beliefs can contribute to error learning from negative emotions; a strong open EMC is not required (van Dyck et al., 2005; Rausch et al., 2017). Regarding small error consequences, we found that more error learning takes place from smaller errors when auditors perceive themselves to work in a blame EMC. This result is in contrast to our prediction and the finding by Stefaniak and Robertson (2010). Further analyses into this unexpected benefit of a blame EMC showed that participants in our sample reported blame EMC perceptions around the mid-point of the scale. We argue that some blame-oriented values and beliefs may create a sense of urgency to learn from smaller errors to prevent their recurrence in the future. For example, Smeets et al. (2022) found that auditors who experienced more error strain (which correlated negatively with a supportive climate for learning from error) were more likely to reflect on their errors in order to learn, in line with arguments by Zhao et al. (2019). We conclude that some blame-oriented values and beliefs within an organization may not be as problematic as previously assumed (Heimbeck et al., 2003; Lei et al., 2016; Metcalfe, 2017; Smeets et al., 2022) and may even carry some potential benefits for error learning. Finally, in post-hoc analyses, we found that time pressure and error learning are related through an inverted-U-shaped function, showing that medium levels of time pressure are associated with more error learning than lower or higher time pressure. This finding is in line with prior research linking time pressure to performance, creativity, and learning in general (e.g., Spilker, 1995; Baer and Oldham, 2006; Prem et al., 2017; Ryari et al., 2021), but is novel in the context of learning from errors, extending earlier findings and conceptualizations.

### 5.1. Theoretical contribution

The current paper contributes to extant research on learning from errors and error management in four principal ways. First, we add to the scarce research on factors that inhibit learning (e.g., Bligh et al., 2018; Grohnert et al., 2019; Zhao et al., 2022) by adding insights on conditions that relate to the error (such as small consequences, routine-type errors, strong negative emotions, and high time pressure) and that may inhibit error learning, as well as to perceptions of the work environment in which error learning takes place (perceived blame EMC). Exploring factors that hinder as well as foster learning from errors can afford a more complete understanding of the learning process (e.g., Lei et al., 2016; Tulis et al., 2016). Second, in this study we explored open and blame EMC perceptions simultaneously as separate concepts, in line with van Dyck et al. (2005). Prior research has predominantly studied the open facet, implicitly inferring information about the blame facet (van Dyck et al., 2005; Klamar et al., 2022; Matthews et al., 2022). Our results show that there is a limited correlation between perceptions of an open and blame EMC—professionals can perceive themselves to work in an open and a blame EMC at the same time. However, the two facets produce distinct, rather than mirrored findings. Consequently, we suggest that both concepts should be measured separately within the same study, and that inferring effects of a blame EMC based on the open EMC scale may not be appropriate. Third, this study is one of the first to explore the interaction between EMC and other conditions for error learning. Our findings add to prior research that has established a direct positive relationship of an open EMC and (error) learning (i.e., Grohnert et al., 2019; Smeets et al., 2022), showing that values and beliefs around learning from error can have a direct as well as a moderating effect in the face of conditions that make learning from errors challenging. Moreover, we add to the limited prior research on these interactions (i.e., Stefaniak and Robertson, 2010; Matthews et al., 2022; Zhao et al., 2022). We therefore propose taking into account perceptions of the work environment as a moderator in studies on factors that drive and/or inhibit error learning. Finally, following prior research that conceptualizes the relationship between time pressure and performance as non-linear (e.g., Spilker, 1995; Baer and Oldham, 2006; Prem et al., 2017; Ryari et al., 2021), we propose that conceptualizations of error learning processes take into account potential non-linear relationships in which both inhibiting conditions and fostering factors are not related to error learning in a straightforward manner. On the one hand, this will add nuance to our insights on error learning; on the other hand, it can inform practice on the degree to which specific mechanisms need to be absent/present for learning to take place.

### 5.2. Practical implications

Following our insights on how professionals’ perceptions of their firm’s EMC interact with the four inhibitors in relation to error learning, we derive implications for organizations, leaders and professionals at all levels. First, organizations may benefit from more explicitly communicating the value of all errors, including those with small consequences to their members, by emphasizing their learning potential, especially given that errors with no or non-severe consequences are fairly common in the workplace. Interestingly, it appears that some blame-based values and beliefs diminish this particular barrier to learning, possibly due to heightened alertness on the part of the error-maker. While we are cautious in recommending the maintenance of a blame climate based on this finding, it does imply that there is some merit in communicating error repercussions to organizational members. Indeed, we also find that auditors reported more error learning accompanying stronger negative emotions when they perceived themselves to work in an open EMC, implying that organizations should seek to strike a balance between components of both open and blame EMCs. Finally, the auditing profession suffers from intense levels of time pressure due to the cyclical nature of the work and tight deadline pressures—as is also the case in other fields. As a result, our finding that excessive time pressure results in lower levels of error learning is particularly concerning. Since time pressure is an inherent feature of many workplaces, organizations must find ways to cope with the resulting threats, such as reduced learning from error. Our results point to the benefits of an open EMC in mitigating this problem.

More generally, following Salancik and Pfeffer’s (1978) social information-processing theory, organizations can only indirectly influence professionals’ individual perceptions. Prior research has shown that auditors’ perceptions of a learning from error climate are driven predominantly by leaders’ behaviors, rather than by official communications and formal structures (Grohnert et al., 2019; Smeets et al., 2021). According to Schein and Schein (2017), leaders influence what is valued and rewarded at work by the way they allocate attention, time, monetary resources, and praise, how they choose to select, mentor, and promote professionals, and how they react to critical incidents (Schein and Schein, 2017). Creating an open EMC then centers on being a role model for learning from one’s own errors, providing opportunities for reporting errors rather than punishing subordinates, listening and assisting in the analysis and mitigation of future errors, and sharing knowledge derived from errors with others (van Dyck et al., 2005; Putz et al., 2012; Grohnert et al., 2019; Gold et al., 2022; Smeets et al., 2022). This requires that leaders have clear expectations for managing errors in their teams, as well as holding team members accountable for creating an open EMC (e.g., Edmondson, 2004; Lupton and Warren, 2018). According to Schein and Schein (2017), the effectiveness of these leader behaviors will depend on whether or not their underlying values and beliefs are also anchored in an organization’s structures, systems, and routines, for example, in selection and promotion decisions (Schein and Schein, 2017). Both mechanisms need to be aligned for the creation and maintenance of a coherent and effective EMC. Additionally, organizations need not strive for a ‘perfect’ EMC—the presence of some blame values in a firm may not be problematic for effective error learning—implementation need not be perfect, but ‘good enough’ to foster learning from smaller errors, with stronger negative emotions and when dealing with time pressure.

### 5.3. Limitations and future research

The results and conclusions presented in this paper should be interpreted in light of the following limitations. First, by using an experiential questionnaire, we relied on auditors’ self-reported perceptions. While we did not find evidence of common method bias in participants’ responses to our instrument, future research can further address this limitation by combining multiple information sources, such as reports by several team members on the same error, or combining perceptions by leaders and team members around a specific critical incident (Flanagan, 1954). Second, our data were collected in a single professional setting, namely, Dutch audit firms. Auditing is a highly relevant setting for research on error learning, and the focused approach limited noise in the data due to standardized certification and professional development, detailed national and international regulation, and high levels of proceduralization. However, this focus also leads to limitations in terms of generalizability. Consequently, future research is needed to establish whether the relationships found in this study translate to other contexts. Specifically, we propose future research to explore both open and blame EMC in the same study. In selecting other contexts, our literature review suggests focusing both on knowledge-intensive fields with team-based work, and on settings with different levels of complexity and time pressure (Matthews et al., 2022; Smeets et al., 2022; Zhao et al., 2022). Third, our sample does not enable us to conduct a multilevel analysis in which perceptions of their firm’s climate are aggregated across participants, in line with common measurements of EMC (van Dyck et al., 2005). In the current study, we consequently relied on individual perceptions in relation to their behaviors, following prior studies on learning climate (i.e., Park and Rothwell, 2009; Eldor and Harpaz, 2016; Grohnert et al., 2019). We suggest that future research designs, where possible, include members across a significant number of organizations and/or organizational units, allowing the exploration of the nested nature of EMC at the group level versus behaviors at the individual level, such as error learning. Finally, we asked participants to recall errors from their own experience. Recall based on the experiential questionnaire method may not be complete or fully representative of the event (Mahajan, 2010; Rausch et al., 2017). We added time between event and recall as a covariate, which was only significantly related to other covariates, not to model variables. We note, however, that more experienced and female auditors reported errors that were further in the past; this might be a starting point for future research to explore values, beliefs, and assumptions as a driver behind expectations about who is ‘permitted’ to make mistakes, providing a more nuanced picture of the role an EMC plays in fostering learning from errors for different groups of employees.

## Data availability statement

The datasets presented in this study can be found in online repositories. The names of the repository/repositories and accession number(s) can be found at: https://doi.org/10.34894/UZOLBE, *via* The Dataverse Project.

## Ethics statement

The studies involving human participants were reviewed and approved by SBE Ethical Review Board, Vrije Universiteit Amsterdam. The patients/participants provided their written informed consent to participate in this study.

## Author contributions

OM, TG, and AG contributed to the conception and design of this study, all authors were involved in the data collection process. OM and TG organized the dataset and performed the statistical analyses. OM wrote the first draft of the manuscript, TG and AG wrote the current version of the manuscript, and engaged in supervision of OM. All authors contributed to the article and approved the submitted version.

## Conflict of interest

The authors declare that the research was conducted in the absence of any commercial or financial relationships that could be construed as a potential conflict of interest.

## Publisher’s note

All claims expressed in this article are solely those of the authors and do not necessarily represent those of their affiliated organizations, or those of the publisher, the editors and the reviewers. Any product that may be evaluated in this article, or claim that may be made by its manufacturer, is not guaranteed or endorsed by the publisher.

## Footnotes

^1^Using all 11 items, we find acceptable sampling adequacy (KMO = 0.794), and variables are not overly correlated to affect the outcome of the PCA (Bartlett’s test of sphericity: *X*^2^(55) = 0.794, *p* = 0.000). Initial analyses revealed three components with eigenvalues above 1, in line with Kaiser’s criterion, explaining a cumulative 57.3% of the variance. The scree plot showed inflections that would justify both a one and a three-component solution. The component plot indicates that nine of the 11 items are closely clustered together, with two items of the error reflection scale separated (“I did not know how to proceed after my error, so I relied on my colleagues” and “I was unable to correct the error by myself, so I turned to my colleagues”), which is likely due to the kind of errors reported by participants. Rerunning the PCA without these two items revealed a one-component solution (KMO = 0.861; Bartlett’s test *p* = 0.000; eigenvalue = 3.661, variance explained = 40.676%).

## Appendix

### Open-ended questions in the experiential questionnaire

Questions used for determining error consequence.

- If applicable, please indicate the impact of the described error for the audit itself (e.g., led to adjustments or additions in audit procedures).
- If applicable, please indicate the impact of the described errors for you personally in terms of upcoming engagement performance evaluations.
- If applicable, please indicate the impact of the described error for people around you in terms of colleagues (subordinates, peers, and superiors) and the client.

Questions used for determining error type.

- With your chosen example in mind, please describe the following: (I) the task you were performing, (II) the error you made during the task.
- Please explain how the described error was discovered.
- What steps did you undertake to handle the described error?
